# Three-dimensional Nitrogen-Doped Graphene Supported Molybdenum Disulfide Nanoparticles as an Advanced Catalyst for Hydrogen Evolution Reaction

**DOI:** 10.1038/srep17542

**Published:** 2015-12-07

**Authors:** Haifeng Dong, Conghui Liu, Haitao Ye, Linping Hu, Bunshi Fugetsu, Wenhao Dai, Yu Cao, Xueqiang Qi, Huiting Lu, Xueji Zhang

**Affiliations:** 1 Beijing Key Laboratory for Bioengineering and Sensing Technology, School of Chemistry & Biological Engineering, University of Science & Technology Beijing, Beijing 100083, P.R. China; 2 School of Engineering and Applied Science, Aston University, Birmingham, B4 7ET, United Kingdom; 3 Chemistry and Chemical Engineering, Chongqing University, No. 174 Shazhengjie, Shaping Ba, Chongqing, 400044, P. R. China; 4 Japan Policy Alternative Research Institute, The University of Tokyo, 2-11-16 Yayoi, Bunkyo-ku, Tokyo 113-0032, Japan

## Abstract

An efficient three-dimensional (3D) hybrid material of nitrogen-doped graphene sheets (N-RGO) supporting molybdenum disulfide (MoS_2_) nanoparticles with high-performance electrocatalytic activity for hydrogen evolution reaction (HER) is fabricated by using a facile hydrothermal route. Comprehensive microscopic and spectroscopic characterizations confirm the resulting hybrid material possesses a 3D crumpled few-layered graphene network structure decorated with MoS_2_ nanoparticles. Electrochemical characterization analysis reveals that the resulting hybrid material exhibits efficient electrocatalytic activity toward HER under acidic conditions with a low onset potential of 112 mV and a small Tafel slope of 44 mV per decade. The enhanced mechanism of electrocatalytic activity has been investigated in detail by controlling the elemental composition, electrical conductance and surface morphology of the 3D hybrid as well as Density Functional Theory (DFT) calculations. This demonstrates that the abundance of exposed active sulfur edge sites in the MoS_2_ and nitrogen active functional moieties in N-RGO are synergistically responsible for the catalytic activity, whilst the distinguished and coherent interface in MoS_**2**_/N-RGO facilitates the electron transfer during electrocatalysis. Our study gives insights into the physical/chemical mechanism of enhanced HER performance in MoS_2_/N-RGO hybrids and illustrates how to design and construct a 3D hybrid to maximize the catalytic efficiency.

Hydrogen has emerged as an effective alternative to fossil fuels because it is environment-friendly energy with water as exhaust^1^. Hydrogen formation from water has long been considered as a promising approach for solar energy storage^2^. To improve the hydrogen transition efficiency, advanced catalysts are continuously explored to reduce the overpotential of the hydrogen evolution reaction (HER), a basic step involved in electrochemical water splitting (2H^+^ + 2e^−^ → H_2_)^2^,^3^,^4^. The widespread application of the most effective catalysts (drawn from the Pt group of metals) are limited by their low natural abundance and high cost^5^. Therefore, there is an urgent need to develop sustainable well-performing alternatives for HER electrocatalysis with high natural abundance and low cost^6^,^7^. Various transition metals including Co, Fe, Ni and their derivatives, and metal-free catalysts like graphitic-carbon nitride coupled with nitrogen-doped graphene have been widely reported for HER^8^,^9^,^10^,^11^,^12^,^13^. However, these non-precious-metal catalysts suffer from instability and low catalytic activity in acidic conditions^13^,^14^. Empirical results and theoretical predications have demonstrated that molybdenum disulphide (MoS_2_) and its derivatives possess high-performance electrocatalytic activity towards HER^15^,^16^. It has been identified that the edge site of MoS_2_ can act as a promising hydrogen evolution catalyst, leading to a lateral dimension size-dependent catalytic activity^5^. However, the deficiencies of aggregation and low conductivity of MoS_2_ are still challenging problems which limit their widespread application. Monolayer metallic MoS_2_ with high conductivity have been explored for HER catalyst^17^,^18^. For example, metallic nanosheets of 1T-MoS_2_ chemically exfoliated via lithium intercalation from semiconducting 2H-MoS_2_ nanostructures grown directly on graphite was reported as a highly competitive earth-abundant catalyst due to favorable kinetics, metallic conductivity, and proliferation of active sites^17^. Meanwhile, the functionalization of MoS_2_ materials with conductive templates or supports to form a multidimensional structure has been recently presented as an effective approach to improve the catalytic activity^19^. Carbon materials are promising candidates to support catalysts due to their unique physicochemical properties. Increasing evidence has demonstrated that the significantly improved HER performance of MoS_2_ can be implemented by incorporating MoS_2_ with carbon nanotube (CNTs), graphene, graphite or other mesoporous carbon to form a MoS_2_-based hybrid or composite^20^,^21^,^22^,^23^,^24^. For example, Dai *et al.* developed an HER advanced catalyst of MoS_2_ nanoparticles grown on graphene via a facile solvothermal approach, the resulted highly exposed edges and excellent electrical coupling to the underlying graphene sheets endowed the hybrid catalyst with excellent HER activity^25^. The high electrical conductance of the carbon architecture facilitates the electron charge transfer leading to enhanced performance, whilst the active site introduced in the supports is another effective method to enhance the activity of the catalyst. The incorporation of electron-rich nitrogen atoms into the carbon architecture promotes the interaction between neighboring carbons and electrons, providing a superior heteroatom-doped catalyst^23^,^26^. Nitrogen-doped graphene is one of the most promising candidates owing to its high chemical stability, good electrical conductivity and intrinsic moderate catalytic activity for hydrogen evolution^27^. For instance, Qiao’s group has obtained porous C_3_N_4_ nanolayers@N-graphene films by integrating porous C_3_N_4_ nanolayers with nitrogen-doped graphene sheets, displaying an unbeatable HER performance which stems from highly exposed active sites, hierarchical porous structure and 3D conductive graphene network^28^. Furthermore, it has been reported that the p-type MoS_2_ nanoplatelets grown on the n-type nitrogen-doped reduced graphene oxide (N-RGO) can form p−n junctions on nanoscale and act as an outstanding catalyst in photocatalytic HER^29^. However, to the best of our knowledge, little work on the influence of the composition and morphology of the nitrogen-doped graphene support to the HER has been reported.

Herein, we report MoS_2_ nanoparticles decorated nitrogen-doped reduced graphene oxide (N-RGO), a three-dimensional (3D) hybrid structure with macro-porosity by a hydrothermal route, which can efficiently catalyze HER under acidic conditions with low overpotential and small Tafel slope. The resulting MoS_2_/N-RGO hybrid materials display high-performance HER catalytic activity. By controlling the elemental composition, electrical conductance and morphology of the as-prepared 3D MoS_2_/N-RGO hybrid, the enhanced electrocatalytic activity mechanism toward hydrogen evolution was investigated in detail.

## Results

### Structural and compositional characterization

**Figure 1:**
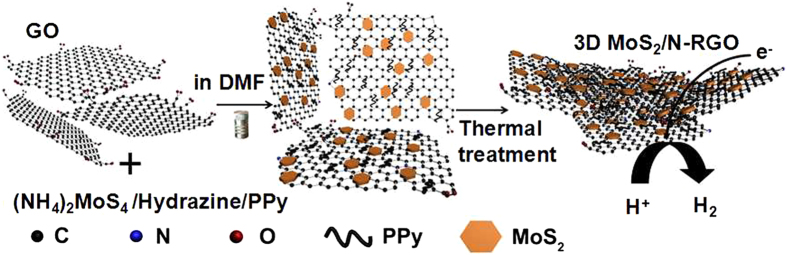
Schematic present of synthetic method and resultant 3D MoS_2_/N-RGO hybrid for hydrogen evolution reaction.

Firstly, graphene oxide (GO) and (NH_4_)_2_MoS_4_ were sonicated in N, N-dimethylformamide (DMF) to generate a highly dispersed suspension. Hydrazine and polypyrrole (PPy) were then slowly added into the suspension, followed by hydrothermal assembly at 180 °C for 12 h. Besides as a reductant, hydrazine could work as a nitrogen source as PPy during the assembly process. Regarding to PPy, it acted not only as the nitrogen source, but also was used to fabricate 3D network structure which promoted the BET specific surface and conductivity of the final product. The hydrothermal environment plays a significant role in the nitrogen doping because in the absence of hydrothermal environment, the GO could be reduced directly without the incorporation of nitrogen into the graphene network. During the hydrothermal process, (NH_4_)_2_MoS_4_ and GO were reduced to MoS_2_ nanoparticles and RGO respectively by hydrazine; and MoS_2_ nanoparticles were grown on the RGO accompanied by incorporation of nitrogen species into the graphene lattice^22^. In order to sustain the perfect 3D structure, the as-prepared hybrid was directly dehydrated by a vacuum freeze drier and then heated at 600 °C for 3 h under nitrogen (Fig. 1). Other samples were also fabricated in the same way for comparison: N-RGO where (NH_4_)_2_MoS_4_ is removed from the starting materials; MoS_2_’/N-RGO where nitrogen-doped graphene sheets decorated with half quantity of MoS_2_; MoS_2_/N’-RGO where the addition order of hydrazine and PPy is exchanged; MoS_2_/N-RGO’ is the sample fabricated by GO of higher oxidation degree.

**Figure 2:**
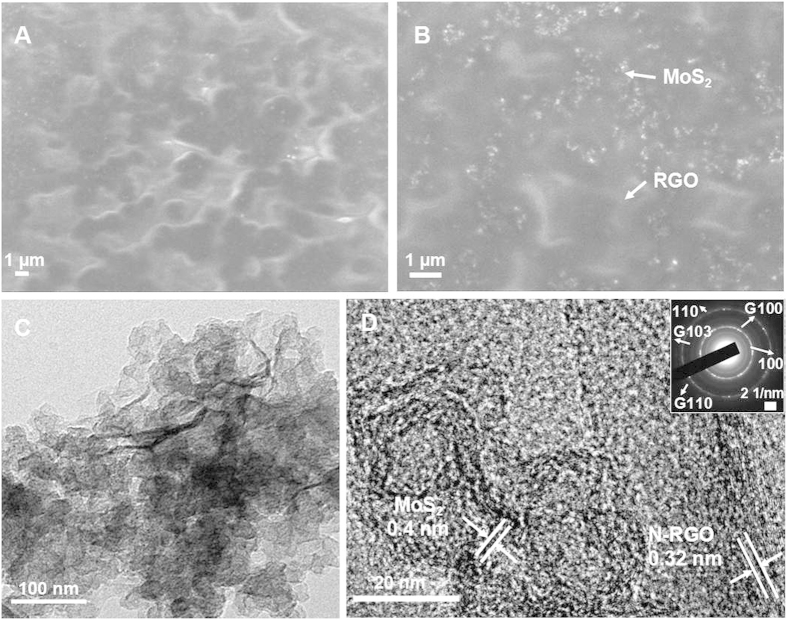
(**A**) SEM and (**B**) magnified SEM images of 3D MoS_2_/N-RGO hybrid. (**C**) TEM images and (**D**) high-resolution TEM images of the 3D MoS_2_/N-RGO hybrid.

The 3D MoS_**2**_/N-RGO synthesized was comprehensively characterized by a number of microscopic and spectroscopic tools. Scanning electron microscopic (SEM) images revealed a 3D network structure with crumpled few-layered graphene sheets with plenty of folded edges conglutinated by PPy and decorated by numerous MoS_2_ nanoparticles with a lateral dimension of about 35 nm (Fig. 2A,B). The large area of graphene provided a large attachment surface for MoS_**2**_nanoparticle growth and created an efficient template to restrain the aggregation of particles. The information presented by the transmission electron microscopic (TEM) images was consistent with the SEM in that there were laminar structures of graphene in the catalyst and MoS_**2**_nanoparticles were evenly distributed on N-RGO sheets, suggesting successful assembly between the nanoparticles and the graphene sheets (Fig. 2C). The high resolution TEM (HRTEM) images of the 3D MoS_**2**_/N-RGO structure showed the MoS_**2**_ with a lattice spacing of 0.4 nm, consistent with the spacing between the two sulfur atoms, which indicates the presence of MoS_**2**_. The thickness of N-RGO was 0.34 nm, in accordance with the theoretical prediction^30^ (Fig. 2D). The corresponding selected area electron diffraction (SAED) revealed several sets of diffraction signals assigned to planes of hexagonal-phase of MoS_2_ and N-RGO (inset in Fig. 2D). The two separated diffraction rings can be indexed to the (100) and (110) planes of MoS_2_, and the diffraction of the (103) assigned to the N-RGO can also be observed. Notably, a distinguished and coherent interface was observed in MoS_**2**_/N-RGO, which is known to facilitate the electron transfer to enhance catalytic activity^30^.

**Figure 3:**
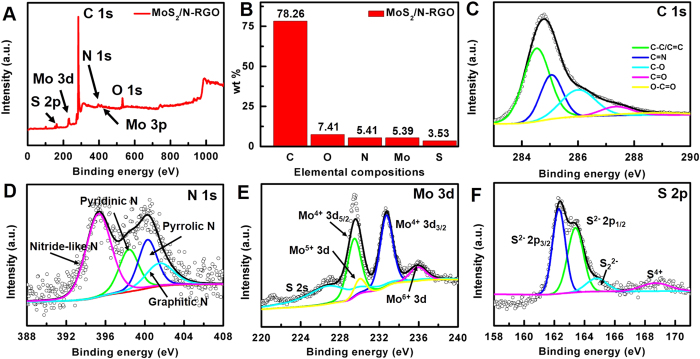
(**A**) The survey XPS spectrum and (**B**) corresponding elemental compositions of 3D MoS_2_/N-RGO catalyst. High-resolution (**C**) C 1 s, (D) N 1 s, (**E**) Mo 3d and (**F**) S 2p XPS spectra of 3D MoS_2_/N-RGO (the red curve: background; the black curve: fitting line; the open circle: raw date).

X-ray photoelectron spectroscopy (XPS) measurements were performed to investigate the components of the prepared 3D MoS_2_/N-RGO hybrid. As shown in Fig. 3A, the XPS survey spectrum of the 3D MoS_2_/N-RGO hybrid shows the characteristic peaks of N-RGO; including a prominent graphitic C 1 s peak at 284 eV, a strong O 1 s peak at 532 eV and an N 1 s peak located at 402 eV. The peaks assigned to Mo 3d and S 2p in MoS_2_ were also present in the survey spectrum, which confirmed the successful assembly between the nanoparticles and the graphene sheets. The detailed information of elemental composition is presented in Fig. 3B. The corresponding element analysis revealed that the atomic ratio of Mo (0.75%) to S (1.46%) was about 1:2, consistent with the theoretical value of MoS_2_. It is worthy mentioning that the N/C atomic ratio of the 3D MoS_2_/N-RGO hybrid was calculated up to be 5.9%, much higher than that of N-doped graphene from previous reports^31^,^32^,^33^. The higher nitrogen element included in the 3D MoS_2_/N-RGO hybrid could lead to a more efficient catalytic activity^30^.

In the high resolution XPS of C 1 s (Fig. 3C), a prominent C = N peak (285.2 eV) can be observed, confirming the successful incorporation of N atoms into the 3D MoS_2_/N-RGO hybrid. The binding energy of C-C/C=C (284.5 eV) in the 3D MoS_2_/N-RGO hybrid was downshifted by ∼0.5 eV compared to the graphitized carbon peak of pristine GO (285.0 eV)^34^. This implies a remarkable amount of charge transfer from graphene to MoS_2_, which can improve both the current density and catalytic activity of the catalyst. The N 1 s region shown in Fig. 3D can be divided into four peaks: (1) the peak at 395.7 ± 0.3 eV is attributed to nitride-like species or aromatic N-imines; the pyridine-like peak located at 398.7 ± 0.3 eV; (3) the pyrrolic or amine moieties peak at 400.3 ± 0.3 eV; and (4) the graphitic nitrogen peak at 401.4 ± 0.3 eV^35^,^36^. The high content of nitride-like peak was attributed to the adsorbed N_2_H_4_^37^. It was reported that hydrazine were reducing reagents^37^, the nitride-like peak located at 395.7 eV should be ascribed to the adsorbed hydrazine, even samples experienced several-times washing and dialysis^37^. PPy was an efficient nitrogen precursor to fabricate nitrogen-doped graphene due to its excellent catalytic activity and high durability, which mainly existed in forms of pyrrolic N (401.0 ± 0.2 eV) and pyridinic N (398.1 ± 0.2 eV) in NGO name^38^. The characteristic peaks of nitride-like peak, pyrrolic and pyridinic N in the resulting hybrid materials indicated that hydrazine and PPy were both nitrogen source.

As shown in Fig. 3E, five characteristic peaks located at 228.8, 232.7, 230.0, 235.9 and 226.7 eV, corresponding to Mo^4+^ 3d_5/2_, Mo^4+^ 3d_3/2_, MoO_3_ or MoO_4_^2−^, Mo^5+^ and S 2 s, respectively, are displayed in the high resolution XPS of Mo 3d^39^. The two main Mo 3d_5/2_ and Mo 3d_3/2_ peaks are close to the meta-stable 1 T phase of MoS_2_ (associating with better HER performance)^40^,^41^, and the peak of Mo^6+^ results from the oxidation of the catalyst in air to form MoO_3_ or MoO_4_^2−^ 34 and the partial reduction of Mo^6+^ results in the formation of Mo^5+^ 39. The deconvolution of S 2p spectra (Fig. 3F) has yielded four main peaks located at 162.3, 163.4, 164.8 and 168.7 eV, which are assigned to S 2p_3/2_, S 2p_1/2_, S_2_^2−^ (or S^2−^) ligands and the S^4+^ of sulfate groups, respectively^40^,^43^,^44^. The existence of S_2_^2−^ and/or S^2−^ is likely to be caused by the doping of S in the graphene sheet or formation of an S-rich MoS_2_ structure. This facilitates the development of high HER activity at the active sites for HER on the edge of MoS_**2**_where the unsaturated S atoms are exposed to adsorb H^7^,^40^,^43^,^44^.

**Figure 4:**
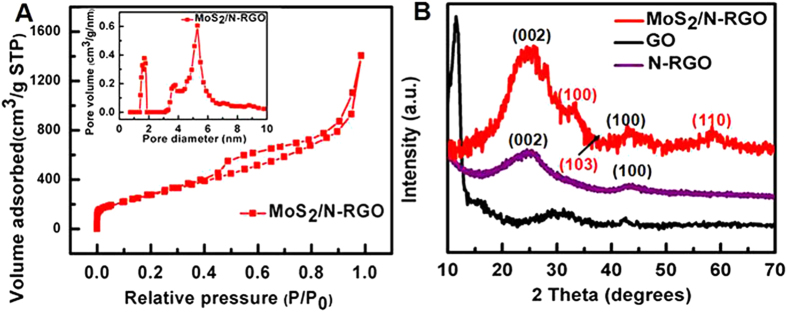
(**A**) Nitrogen adsorption-desorption isotherm and pore-size distribution (inset) of 3D MoS_2_/N-RGO. (**B**) XRD patterns of GO, N-RGO and 3D MoS_2_/N-RGO.

The porosity structures of the 3D MoS_**2**_/N-RGO hybrid were characterized using the nitrogen isotherm (Fig. 4A). The N_2_ adsorption-desorption isotherm of 3D MoS_**2**_/N-RGO hybrid showed an intermediate shape between types II and IV (in the IUPAC classification) with a small H3 hysteresis loop extending from P/P_0_ = 0.45 to 0.95, demonstrating the presence of mesoporous structure and slit-shaped pores^45^. The N_2_ adsorption-desorption measurement revealed the as-prepared 3D MoS_**2**_/N-RGO hybrid had a Brunauer-Emmett-Teller (BET) specific surface area of 1066.6 m^2^ g^−1^, which was significantly larger than many reported porous carbon nanomaterials^46^,^47^,^48^,^49^. The superior BET specific surface area was resulted from that unique 3D structure of MoS_2_/N-RGO hybrid materials and formation of numerous porous structures during the sintering and elimination process in the presence of PPy. The pore-size distribution of the 3D MoS_**2**_/N-RGO hybrid calculated by the DFT method exhibited one dominant peak at 5.30 nm and another two weak peaks at ∼1.56 and 3.79 nm, respectively (Inset of Fig. 4A), suggesting that micropores and mesopores co-exist within the 3D MoS_**2**_/N-RGO hybrid. Figure 4B showed XRD patterns of the 3D MoS_**2**_/N-RGO hybrid, N-RGO and GO. The sharp oxidation peak at 11.7° distinctly exhibited in GO but had completely disappeared in MoS_**2**_/N-RGO and N-RGO. However, for the latter a broad peak centered at around 24° which was associated with a graphitic crystal structure, implying that the GO was efficiently deoxidized during the hydrothermal process. In comparison with N-RGO, the diffraction peaks at 33.5°, 39.4° and 58.9° corresponding to (100), (103) and (110) of MoS_2_ could be observed in MoS_**2**_/N-RGO, indicating the MoS_2_ nanoparticles have been successfully grown on the N-RGO surface. The disappearance of the typical (002) peak of MoS_**2**_located at 14.1° resulted from the small size of the MoS_**2**_ nanoparticles preferring to attach to the surface of the silicon supports^50^,^51^,^52^.

**Figure 5:**
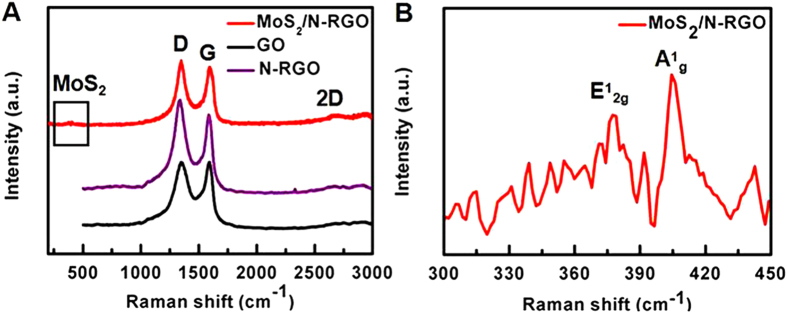
(**A**) Raman spectra of 3D MoS_2_/N-RGO, GO and N-RGO. (**B**) Raman spectra of 3D MoS_2_/N-RGO in the range of 300–450 cm^−1^.

The successful fabrication of the 3D MoS_**2**_/N-RGO hybrid was further confirmed by Raman spectra analysis. As shown in Fig. 5A, the characteristic peaks located at ~1346 and 1599 cm^−1^ associated with the D and G bands of graphene can be observed in GO, N-RGO and MoS_2_/N-RGO. The reduction of GO induces the recovery of the graphene structure, which leads to the increase of 2D bands in N-RGO and MoS_2_/N-RGO. Notably, compared to the GO with a G band at 1599 cm^−1^, the G band of N-RGO (1586 cm^−1^) and MoS_2_/N-RGO (1593 cm^−1^) showed a downshift due to the incorporation of N heteroatoms^49^. The characteristic peaks of MoS_2_ located at 375.5 and 403.5 cm^−1^ (and assigned to the in-plane E^1^_2g_ and out-of-plane A^1^_g_ vibrational modes of the hexagonal MoS_2_) were observed in the Raman spectrum of MoS_2_/N-RGO ranging from 300 to 450 cm^−1^ (Fig. 5B)^53^. In comparison with MoS_2_, the decreasing difference between the peak frequencies of E^1^_2g_ and A^1^_g_ for MoS_2_/N-RGO (27 cm^−1^) suggested that the interaction between the Mo precursors and graphene could efficiently avoid the aggregation of MoS_2_^39^.

### Electrochemical activity and durability

**Figure 6:**
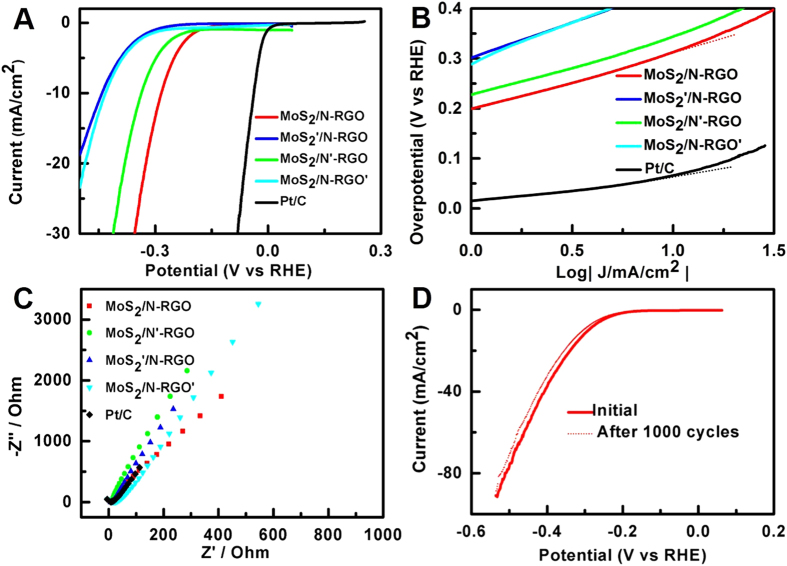
(**A**) Polarization curves obtained of catalysts as indicated and (**B**) corresponding Tafel plots recorded on glassy carbon electrodes with a catalyst loading of 0.28 mg/cm^2^ (solid lines), and fitted Tafel plots (dashed dot). (**C**) Nyquist plots of the 3D MoS_2_/N-RGO catalyst recorded in nitrogen-purged 0.5 M H_2_SO_4_ from 10^−2^ to 10^6^ Hz with an AC amplitude of 5 mV. (**D**) Stability test for the 3D MoS_2_/N-RGO catalyst. Negligible current was lost after 1000 cycles from −0.2 to −0.8 V at 10 mV/s.

The electrocatalytic activity of the as-prepared 3D MoS_**2**_/N-RGO hybrid toward the HER was investigated in 0.5 M H_**2**_SO_**4**_ solution with commercial Pt catalyst (10 wt % Pt on carbon black) as the reference. N-RGO, MoS_2_’/N-RGO, MoS_2_/N-RGO, MoS_2_/N’-RGO and MoS_2_/N-RGO’ were also prepared to investigate in detail the influence of composition and structure to the HER catalytic activity. XPS (Fig. S1), XRD (Fig. S2) and SEM (Fig. S3) confirmed the successful synthesis of the three catalysts. As shown in Fig. 6A, the polarization curves revealed that the as-generated 3D MoS_**2**_/N-RGO displayed a small onset potential of 112 mV for HER activity, after which the cathodic current rose rapidly. In contrast, the MoS_2_’/N-RGO (210 mV), MoS_2_/N’-RGO (155 mV) and MoS_2_/N-RGO’ (200 mV) showed more negative onset potential, indicating lower HER activity. The current intensity of the 3D MoS_**2**_/N-RGO hybrid was higher than the other catalysts along the whole potential region. To further investigate the HER activity, the linear portions of the Tafel plots were fitted to the Tafel equation (η = a + b log |j|), where j is the current density and b is the Tafel slope. In Fig. 6B, the generated MoS_**2**_/N-RGO hybrid showed a Tafel slope of 44 mV per decade, which outperformed MoS_2_’/N-RGO of 114 mV per decade, MoS_2_/N’-RGO of 71 mV per decade and MoS_2_/N-RGO’ of 57 mV per decade. The slight higher Tafel slope compared to MoS_2_ nanoparticle on graphene with a value of 41 mV^25^ and metallic MoS_2_ nanosheets on graphite with a value of 43 mV^17^ may be resulted from the reduce of the conductivity of nitrogen dopants. Notably, the lower HER activity of other catalysts prepared led to higher Tafel slopes than the MoS_**2**_/N-RGO hybrid. Electrochemical impedance spectroscopy (EIS) was measured to evaluate the HER catalytic activity of these catalysts. The MoS_**2**_/N-RGO displayed the lowest faradaic impedance among these prepared catalysts (Fig. 6C) and was comparable with Pt/C, suggesting a small charge transfer resistance in the MoS_**2**_/N-RGO^54^. Such a low charge transfer resistance resulted both from a distinguished and coherent interface between the MoS_**2**_/N-RGO hybrid and from the good conductivity of N-RGO, leading to efficient electrical communication between the catalytic edge sites and underlying electrodes to facilitate the kinetic response of HER^55^,^56^. Stability is a significant criterion to evaluate the practicality of a HER catalyst. After a long period of 1000 potential-cycling between −0.4 and 0 V, the as-generated MoS_**2**_/N-RGO hybrid exhibited a negligible decrease in the current density, indicating outstanding electrochemical stability (Fig. 6D). The unique three-dimensional structure of the MoS_**2**_nanoparticles grown on N-doped graphene may account for the good stability that was observed. The supports of the N-doped graphene restrain the agglomeration of MoS_**2**_nanoparticles and facilitate the transport of electrolyte ions^56^,^57^. In addition, the intercalation of sulfur into the graphene structure to form a stable covalent bond also contributes to the excellent stability (Fig. 3F).

## Discussion

These results demonstrated that the 3D MoS_**2**_/N-RGO hybrid exhibited superior electrochemical catalytic activity to MoS_**2**_’/N-RGO, MoS_**2**_/N’-RGO and MoS_**2**_/N-RGO’, the reasons for the enhanced mechanism of MoS_**2**_/N-RGO hybrid toward HER activity can be explained as followed. Firstly, structure analysis derived from Nitrogen Sorption date (Fig. S4) showed that the 3D MoS_**2**_/N-RGO hybrid had an 87% mesoporous surface area ratio (the mesopore surface area to BET total surface area), which was larger than MoS_2_’/N-RGO and MoS_2_/N’-RGO, which lead to better reactant accessibility and superior efficiency of build of the triple-phase boundaries (gas–electrode–electrolyte)^58^,^59^. Secondly, both of the N-RGO without decorated MoS_**2**_ (Fig. S5) and MoS_**2**_’/N-RGO with less decorated MoS_**2**_ (Fig. 6A) displayed a little HER activity compared to the proposed MoS_**2**_/N-RGO hybrid, indicating the composition of MoS_**2**_nanoparticles played a critical role in the active HER catalytic performance^13^.

**Figure 7:**
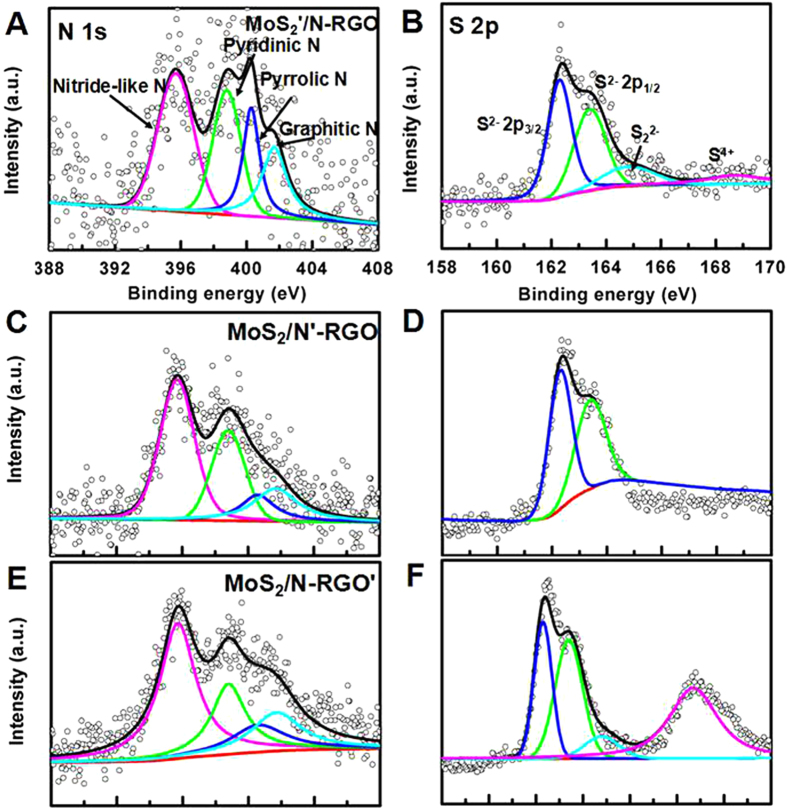
High-resolution XPS spectra of (A), (C), (E) N 1 s and (B), (D), (F) S 2p of the MoS_2_’/N-RGO, MoS_2_/N’-RGO and MoS_2_/N-RGO’ (the red curve: background; the black curve: fitting line; the open circle: raw date).

**Figure 8:**
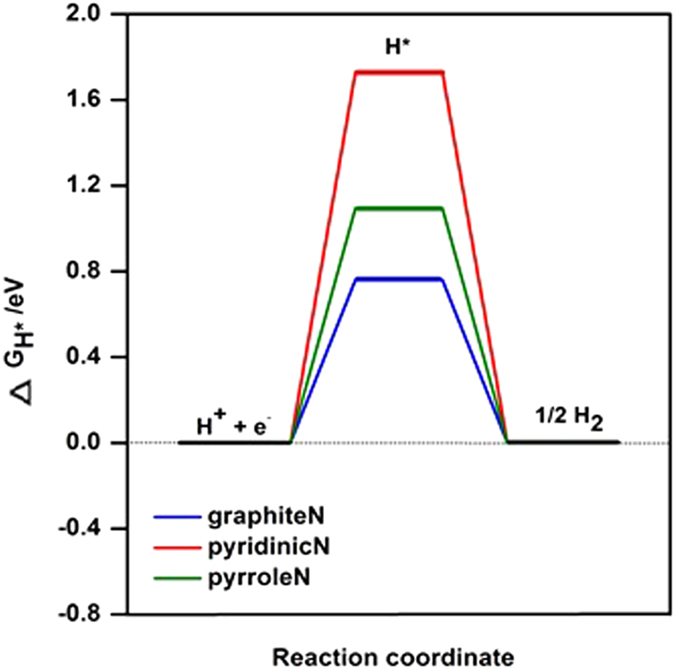
The calculated free-energy diagram of HER for graphite N, pyridinic N and pyrrole N catalysts.

Furthermore, the proposed hybrid contains effective catalytic sites originated from sulfur in MoS_2_ and nitrogen impurities in RGO boost the activity of the catalyst. The relative content (RC) of S_2_^2−^ and/or apical S^2−^related to active HER catalytic activity, where RC is mean that sulfur elemental compositions (wt %) (Table S1) multiply sulfur distribution (at. %) (Table S2), in MoS_**2**_/N-RGO hybrid is 3.5-folds and 1.9-folds higher than MoS_**2**_’/N-RGO (Fig. 7B), MoS_**2**_/N-RGO’ (Fig. 7F), while the distribution of S_2_^2−^ and/or apical S^2−^ in MoS_**2**_/N’-RGO (Fig. 7) was not observed. For the nitrogen impurities, it was found the nitrogen hybrid species are sensitive to the graphene oxidation degree, MoS_2_, PPy and hydrazine (Fig. 7). The MoS_**2**_/N-RGO hybrid contains more pyrrolic N and less pyridinic N than MoS_**2**_’/N-RGO, MoS_**2**_/N’-RGO and MoS_**2**_/N-RGO’ (Fig. 7A,C,E, Table S3), it was speculated that the pyrrolic N instead of pyridinic N is more active towards HER catalysis. The overall HER pathway can be described by a three-state diagram comprising an initial state H^+^ + e^−^, an intermediate adsorbed H*, and a final product 1/2H_2_^60^. The Gibbs free-energy of the intermediate state, |∆G_H*_|, has been considered as a major descriptor of the HER activity for a wide variety of metal catalysts. The optimum value of |∆G_H*_| should be zero. Density Functional Theory (DFT) calculations showed that graphite N shows the smallest Gibbs free-energy (|∆G_H*_|) value of 0.76 eV, followed by pyrrole N of 1.10 eV and pyridinic N of 1.73 eV, indicating graphite N was the best electrocatalytic activity from the viewpoint of thermodynamics and pyrrolic N also possessed the enhanced electrocatalytic activity than pyridinic N toward HER (Fig. 8). Additionally, the higher oxidation degree of the RGO’ can induce the more nitrogen hybrid (Table S1), larger mesopore surface area but larger faradaic impedance (Fig. 6C), which led to a lower HER performance. Therefore, it was demonstrated that the efficient active nitrogen and sulfur species combined with the good conductance and large mesoporous surface area ratio of 3D MoS_**2**_/N-RGO hybrid were all beneficial to the enhanced HER catalytic activity.

## Conclusion

In summary, we have developed an efficient hydrothermal route to successfully synthesize a 3D hybrid material of nitrogen-doped graphene oxide sheets-supported molybdenum disulfide nanoparticles with high capability of hydrogen evolution. The morphology and element of the as-generated 3D MoS_**2**_/N-RGO hybrid were characterized by comprehensively microscopic and spectroscopic methods including SEM, TEM, HRTEM, BET, XRD, XPS and Raman spectroscopy. Electrochemical characterization data including low onset potential, small Tafel slope, low charge transfer resistance and high electrochemical stability revealed the high performance of the MoS_**2**_/N-RGO hybrid toward HER catalytic activity compared to analogous composites. Detailed experimental analysis implies that the abundant active S_2_^2−^ and/or S^2−^ ligand species and pyrrolic nitrogen and graphene N of the 3D MoS_**2**_/N-RGO hybrid and its good conductance were all beneficial to its enhanced HER catalytic activity. The exploration of the enhanced HER mechanism of the MoS_2_/N-RGO hybrid provides guidelines to design and construct 3D hybrids to maximize their catalytic efficiency.

## Methods

### Materials synthesis

Graphene oxide (GO, made from graphite flake, GO’, small flakes and dry platelets) was obtained from XF NANO, INC and graphene supermarket, respectively. Hydrazine monohydrate (N_2_H_4_·H_2_O, 80%) was purchased from Guangdong Guanghua Sci-Tech Co., Ltd (JHD). (NH_4_)_2_MoS_4_, Polypyrrole (PPy,) and Pt/C (10% Pt) were obtained from Sigma-Aldrich. N, N-dimethylformamide (DMF, ≥99.5%) and KOH were purchased from Sinopharm Chemical Reagent Co., Ltd. Sulfuric acid (H_2_SO_4_, 95–98%) and ethanol (99.9%) was acquired from Beijing Chemical Works. All aqueous solutions were prepared with doubly distilled water.

### Materials preparation

22 mg of (NH_4_)_2_MoS_4_ and 10 mg of GO was dispersed in 10 ml of DMF by 10 min of sonication at room temperature. Then 0.1 ml of N_2_H_4_•H_2_O was added to the above solution followed by 30 min of sonication. 100 mg PPy was then added, followed by sonication for 10 min to form a stable complex solution. The mixture solution was transferred to a 40 mL Teflon-lined autoclave and heated in an oven at 180 °C for 12 h with no intentional control of ramping or cooling rate. The product was collected by centrifugation at 10000 rpm for 5 min and washed with DI water several times to remove most of the DMF. Subsequently, the product was redispersed in 3 ml of DI water and freeze-dried overnight, followed by thermal treatment at 600 °C for 3 h in N_2_ gas with 400 standard cubic centimeters per minute (sccm) to remove the organic species (DMF) and improving crystallinity. The obtained product was denoted as 3D MoS_2_/N-RGO. Other samples were also fabricated in the same way for comparison: N-RGO where (NH_4_)_2_MoS_4_ is removed from the starting materials; MoS_2_’/N-RGO where Nitrogen-doped graphene sheets decorated with half quantity of MoS_2_; MoS_2_/N’-RGO where the addition order of hydrazine and PPy is exchanged; MoS_2_/N-RGO’ is the sample fabricated by GO of higher oxidation degree.

### Characterizations

X-ray diffraction (XRD) was performed with a Rigaku X-ray diffractometer with Cu KR target. The porosity was measured with a nitrogen adsorption-desorption isotherm using a surface area analyzer (QuadraSorb SI 2000-08, Quantachrome Instruments). The structure of products was observed under a field-emission scanning electron microscope (SEM; JEOL-6300 F, 3 kV) and a transmission electron microscope (TEM; JEM-2010, 200 kV). X-ray photoelectron spectroscopy (XPS) spectra were obtained using an AXIS ULTRA^DLD^ instrument equipped with an Al Kα X-ray source. Raman spectrum of powder samples were recorded on an InVia-Reflex Raman microscope with a laser excitation wavelength of 532 nm.

### Electrochemical measurement

All electrochemical studies were performed using a CHI 852C electrochemical workstation (Shanghai Chenhua Instrument Co., China) in a standard three-electrode setup. A three-electrode configuration consisting of a saturated calomel electrode (SCE) as the reference electrode, a graphite rod as the counter electrode and a glass carbon RDE after loading the catalyst as the working electrode was employed. Typically, 0.5 mg of catalyst was dispersed in 500 μl of DI water by sonication to form a homogeneous ink. Then, 20 μl of the catalyst ink (containing 20 μg of catalyst) was loaded onto a glassy carbon electrode (GCE) of 3 mm in diameter (loading ~0.283 mg/cm^2^). After the catalyst ink dried, 5 μl of 1 wt% Nafion solution was dropped onto the GCE and the working electrode was prepared. Liner sweep voltammetry (LSV) was conducted in nitrogen-purged 0.5 M H_2_SO_4_ at a scan rate of 3 mV s^−1^ at 1400 rpm. Electrochemical impedance spectroscopy (EIS) was carried out in the same configuration in nitrogen-purged 0.5 M H_2_SO_4_ from 10^−2^ to 10^6^ Hz with a modulation amplitude of 5 mV. SCE was calibrated with respect to reversible hydrogen electrode (RHE). The calibration was performed in the high purity H_2_ saturated electrolyte with a Pt wire as the working electrode and the counter electrode. LSV were run at a scan rate of 0.1 mV s^−1^, and the potential at which the current crossed zero was taken to be the thermodynamic potential for the hydrogen electrode reactions. In 0.5 M H_2_SO_4_, E (RHE) = E (SCE) + 0.314 V. All the potentials reported in our manuscript are against RHE.

### Density Functional Theory (DFT) calculations

The computations for DFT calculations were performed using the *ab initio* density functional code VASP with the PBE exchange correlation functional and PAW potentials. The PBE functional was chosen in order to obtain reasonable adsorption energies. The Brillouin zone is sampled using a 1 × 1 × 1 Monkhorst-Pack grid for the geometry optimizations and a 9 × 9 × 1 Monkhorst-Pack grid for the calculations of electronic properties. A basis set with the cut-off energy of 400 eV was chosen. The convergence criteria for energies and forces are set to 1.0 × 10^−4^ eV and −0.05 eV/Å, respectively. All calculations were performed using spin unrestricted method.

## Additional Information

**How to cite this article**: Dong, H. *et al.* Three-dimensional Nitrogen-Doped Graphene Supported Molybdenum Disulfide Nanoparticles as an Advanced Catalyst for Hydrogen Evolution Reaction. *Sci. Rep.* **5**, 17542; doi: 10.1038/srep17542 (2015).

## Supplementary Material

Supplementary Information

## References

[b1] Joshi A. S., Dincer I. & Reddy B. V. Exergetic assessment of solar hydrogen production methods. Int. J. Hydrogen Energy 35, 4901–4908 (2010).

[b2] Jiao Y., Zheng Y., Jaroniec M. & Qiao S. Z. Design of electrocatalysts for oxygen- and hydrogen-involving energy conversion reactions. Chem. Soc. Rev. 44, 2060–2086 (2015). 25672249 10.1039/c4cs00470a

[b3] Turner J. A. Sustainable hydrogen production. Science 305, 972–974 (2004). 15310892 10.1126/science.1103197

[b4] Walter M. G. *et al.* Solar water splitting cells. Chem. Rev. 110, 6446–6473 (2010). 21062097 10.1021/cr1002326

[b5] Faber M. S. & Jin S. Earth-Abundant inorganic electrocatalysts and their nanostructures for energy conversion applications. Energy Environ. Sci. 7, 3519–3542 (2014).

[b6] Xu S., Li D. & Wu P. One‐pot, facile, and versatile synthesis of monolayer MoS_2_/WS_2_ quantum dots as bioimaging probes and efficient electrocatalysts for hydrogen evolution reaction. Adv. Funct. Mater. 25, 1127–1136 (2015).

[b7] Norskov J. K., Bligaard T., Rossmeisl J. & Christensen C. H. Towards the computational design of solid catalysts. Nature Chem. 1, 37–46 (2009). 21378799 10.1038/nchem.121

[b8] Du P. & Eisenberg R. Catalysts made of earth-abundant elements (Co, Ni, Fe) for water splitting: recent progress and future challenges. Energy Environ. Sci. 5, 6012–6021 (2012).

[b9] Huang Z. *et al.* Cobalt phosphide nanorods as an efficient electrocatalyst for the hydrogen evolution reaction. Nano Energ. 9, 373–382 (2014).

[b10] Mellmann D. *et al.* Base-free non-noble-metal-catalyzed hydrogen generation from formic acid: scope and mechanistic insights. Chem.-Eur. J. 20, 13589–13602 (2014). 25196789 10.1002/chem.201403602

[b11] Borg S. J. *et al.* Electron transfer at a dithiolate-bridged diiron assembly: electrocatalytic hydrogen evolution. J. Am. Chem. Soc. 126, 16988–16999 (2004). 15612737 10.1021/ja045281f

[b12] Dempsey J. L., Brunschwig B. S., Winkler J. R. & Gray H. B. Hydrogen evolution catalyzed by cobaloximes. Accounts. Chem. Res. 42, 1995–2004 (2009). 10.1021/ar900253e 19928840

[b13] Zheng Y. *et al.* Hydrogen evolution by a metal-free electrocatalyst. Nat. Commun. 5, 1–8 (2014). 10.1038/ncomms4783 24769657

[b14] Deng J. *et al.* Highly active and durable non-precious-metal catalysts encapsulated in carbon nanotubes for hydrogen evolution reaction. Energy Environ. Sci. 7, 1919–1923 (2014).

[b15] Morales-Guio C. G., Stern L.-A. & Hu X. Nanostructured hydrotreating catalysts for electrochemical hydrogen evolution. Chem. Soc. Rev. 43, 6555–6569 (2014). 24626338 10.1039/c3cs60468c

[b16] Jaramillo T. F. *et al.* Identification of active edge sites for electrochemical H_2_ evolution from MoS_2_ nanocatalysts. Science 317, 100–102 (2007). 17615351 10.1126/science.1141483

[b17] Lukowski M. A. *et al.* Enhanced hydrogen evolution catalysis from chemically exfoliated metallic MoS_2_ nanosheets. J. Am. Chem.Soc. 135, 10274–10277 (2013). 23790049 10.1021/ja404523s

[b18] Voiry D. *et al.* Conducting MoS_2_ nanosheets as catalysts for hydrogen evolution reaction. Nano Letters 13, 6222–6227 (2013). 24251828 10.1021/nl403661s

[b19] Yan Y., Xia B., Xu Z. & Wang X. Recent development of molybdenum sulfides as advanced electrocatalysts for hydrogen evolution reaction. ACS Catal. 4, 1693–1705 (2014).

[b20] Li D. J. *et al.* Molybdenum sulfide/N-doped CNT forest hybrid catalysts for high-performance hydrogen evolution reaction. Nano Lett. 14, 1228–1233 (2014). 24502837 10.1021/nl404108a

[b21] Chen S., Duan J., Tang Y., Jin B. & Qiao S. Z. Molybdenum sulfide clusters-nitrogen-doped graphene hybrid hydrogel film as an efficient three-dimensional hydrogen evolution electrocatalyst. Nano Energ. 11, 11–18 (2015).

[b22] Pumera M., Ambrosi A. & Chng E. L. K. Impurities in graphenes and carbon nanotubes and their influence on the redox properties. Chem. Sci. 3, 3347–3355 (2012).

[b23] Bian X. *et al.* Nanocomposite of MoS_2_ on ordered mesoporous carbon nanospheres: a highly active catalyst for electrochemical hydrogen evolution. Electrochem. Commun. 22, 128–132 (2012).

[b24] Fang Y. *et al.* A low-concentration hydrothermal synthesis of biocompatible ordered mesoporous carbon nanospheres with tunable and uniform size. Angew. Chem., Int. Ed. 49, 7987–7991 (2010). 10.1002/anie.201002849 20839199

[b25] Li Y. G. *et al.* MoS_2_ nanoparticles grown on graphene: an advanced catalyst for the hydrogen evolution reaction. J. Am. Chem. Soc. 133, 7296–7299 (2011). 21510646 10.1021/ja201269b

[b26] Wang Y. & Jiang X. Facile preparation of porous carbon nanosheets without template and their excellent electrocatalytic property. ACS Appl. Mater. Inter. 5, 11597–11602 (2013). 10.1021/am402669y 24187942

[b27] Wang H., Maiyalagan T. & Wang X. Review on recent progress in nitrogen-doped graphene: synthesis, characterization, and its potential applications. ACS Catal. 2, 781–794 (2012).

[b28] Duan J., Chen S., Jaroniec M. & Qiao S. Z. Porous C_3_N_4_ Nanolayers@ N-Graphene Films as Catalyst Electrodes for Highly Efficient Hydrogen Evolution. ACS Nano 9, 931–940 (2015). 25559360 10.1021/nn506701x

[b29] Maitra U. *et al.* Highly effective visible-light-induced H_2_ generation by single-layer 1T-MoS_2_ and a nanocomposite of few-layer 2H-MoS_2_ with heavily nitrogenated graphene. Angew Chem. Int Ed 125, 13295–13299 (2013). 10.1002/anie.201306918 24218187

[b30] Hou Y. *et al.* A 3D hybrid of layered MoS_2_/nitrogen-doped graphene nanosheet aerogels: an effective catalyst for hydrogen evolution in microbial electrolysis cells. J. Mater. Chem. A 2, 13795–13800 (2014).

[b31] Qu L., Liu Y., Baek J.-B. & Dai L. Nitrogen-doped graphene as efficient metal-free electrocatalyst for oxygen reduction in fuel cells. ACS Nano 4, 1321–1326 (2010). 20155972 10.1021/nn901850u

[b32] Wang Y., Shao Y., Matson D. W., Li J. & Lin Y. Nitrogen-doped graphene and its application in electrochemical biosensing. ACS Nano 4, 1790–1798 (2010). 20373745 10.1021/nn100315s

[b33] Jeong H. M. *et al.* Nitrogen-doped graphene for high-performance ultracapacitors and the importance of nitrogen-doped sites at basal planes. Nano Lett. 11, 2472–2477 (2011). 21595452 10.1021/nl2009058

[b34] Koroteev V. O. *et al.* Charge transfer in the MoS_2_/carbon nanotube composite. J. Phys.Chem. C 115, 21199–21204 (2011).

[b35] Stankovich S. *et al.* Synthesis of graphene-based nanosheets via chemical reduction of exfoliated graphite oxide. Carbon 45, 1558–1565 (2007).

[b36] Zalan Z., Lazar L. & Fulop F. Chemistry of hydrazinoalcohols and their heterocyclic derivatives. part 1. synthesis of hydrazinoalcohols. Curr. Org. Chem. 9, 357–376 (2005).

[b37] Long D. *et al.* Preparation of nitrogen-doped graphene sheets by a combined chemical and hydrothermal reduction of graphene oxide. Langmuir. 26, 16096–16102 (2010). 20863088 10.1021/la102425a

[b38] Wu Z. S. *et al.* 3D nitrogen-doped graphene aerogel-supported Fe_3_O_4_ nanoparticles as efficient electrocatalysts for the oxygen reduction reaction. J. Am. Chem. Soc. 134, 9082–9085 (2012). 22624986 10.1021/ja3030565

[b39] Zheng X. *et al.* Space-confined growth of MoS_2_ nanosheets within graphite: the layered hybrid of MoS_2_ and graphene as an active catalyst for hydrogen evolution reaction. Chem. Mater. 26, 2344–2353 (2014).

[b40] Zhao X., Zhua H. & Yang X. Amorphous carbon supported MoS_2_ nanosheets as effective catalysts for electrocatalytic hydrogen evolution. Nanoscale 6, 10680–10685 (2014). 25089653 10.1039/c4nr01885k

[b41] Zhu H. *et al.* Probing the unexpected behavior of aunps migrating through nanofibers: a new strategy for the fabrication of carbon nanofiber-noble metal nanocrystal hybrid nanostructures. J. Mater. Chem. A 2, 11728–11741 (2014).

[b42] Zhan Y., Liu Z., Najmaei S., Ajayan P. M. & Lou J. Large-area vapor-phase growth and characterization of MoS_2_ atomic layers on a SiO_2_ substrate. Small 8, 966–971 (2012). 22334392 10.1002/smll.201102654

[b43] Benck J. D., Chen Z., Kuritzky L. Y., Forman A. J. & Jaramillo T. F. Amorphous molybdenum sulfide catalysts for electrochemical hydrogen production: insights into the origin of their catalytic activity. ACS Catal. 2, 1916–1923 (2012).

[b44] Zhou W. *et al.* MoO_2_ nanobelts@nitrogen self-doped MoS_2_ nanosheets as effective electrocatalysts for hydrogen evolution reaction. J. Mater. Chem. A 2, 11358–11364 (2014).

[b45] Tao Y. S., Kanoh H., Abrams L. & Kaneko K. Mesopore-modified zeolites: preparation, characterization, and applications. Chem. Rev. 106, 896–910 (2006). 16522012 10.1021/cr040204o

[b46] Liang H. W., Wei W., Wu Z. S., Feng X. & Mullen K. Mesoporous metal-nitrogen-doped carbon electrocatalysts for highly efficient oxygen reduction reaction. J. Am. Chem. Soc. 135, 16002–16005 (2013). 24128393 10.1021/ja407552k

[b47] Pachfule P., Dhavale V. M., Kandambeth S., Kurungot S. & Banerjee R. Porous-organic-framework-templated nitrogen-rich porous carbon as a more proficient electrocatalyst than Pt/C for the electrochemical reduction of oxygen. Chem.-Eur. J. 19, 974–980 (2013). 23203910 10.1002/chem.201202940

[b48] Chen L.F. *et al.* Synthesis of nitrogen-doped porous carbon nanofibers as an efficient electrode material for supercapacitors. ACS Nano 6, 7092–7102 (2012). 22769051 10.1021/nn302147s

[b49] Liu G., Li X., Ganesan P. & Popov B. N. Development of non-precious metal oxygen-reduction catalysts for PEM fuel cells based on n-doped ordered porous carbon. Appl. Catal. B-Environ. 93, 156–165 (2009).

[b50] Zeng Z. *et al.* Single-layer semiconducting nanosheets: high-yield preparation and device fabrication. Ange. Chem., Int. Ed. 50, 11093–11097 (2011). 10.1002/anie.201106004 22021163

[b51] Matte H. S. S. R. *et al.* MoS_2_ and WS_2_ analogues of graphene. Ange. Chem., Int. Ed. 49, 4059–4062 (2010). 10.1002/anie.201000009 20425874

[b52] Lin Z., Waller G., Liu Y., Liu M. & Wong C.-P. Facile synthesis of nitrogen-doped graphene via pyrolysis of graphene oxide and urea, and its electrocatalytic activity toward the oxygen-reduction reaction. Adv. Energy Mater. 2, 884–888 (2012).

[b53] Kong D. *et al.* Synthesis of MoS_2_ and MoSe_2_ films with vertically aligned layers. Nano Lett. 13, 1341–1347 (2013). 23387444 10.1021/nl400258t

[b54] Hou Y., Zuo F., Dagg A. P., Liu J. & Feng P. Branched WO_3_ nanosheet array with layered C_3_N_4_ heterojunctions and CoO_x_ nanoparticles as a flexible photoanode for efficient photoelectrochemical water oxidation. Adv. Mater. 26, 5043–5049 (2014). 24848321 10.1002/adma.201401032

[b55] Hou Y. *et al.* Metal-organic framework-derived nitrogen-doped core-shell-structured porous Fe/Fe_3_C@C nanoboxes supported on graphene sheets for efficient oxygen reduction reactions. Adv. Energy Mater. 4, 1400337–1400344 (2014).

[b56] Yang S., Feng X., Ivanovici S. & Muellen K. Fabrication of graphene-encapsulated oxide nanoparticles: towards high-performance anode materials for lithium storage. Ange. Chem., Int. Ed. 49, 8408–8411 (2010). 10.1002/anie.201003485 20836109

[b57] Chen D., Ji G., Ma Y., Lee J. Y. & Lu J. Graphene-encapsulated hollow Fe_3_O_4_ nanoparticle aggregates as a high-performance anode material for lithium ion batteries. ACS Appl. Mater. Inter. 3, 3078–3083 (2011). 10.1021/am200592r 21749101

[b58] Park K. W. *et al.* New RuO_2_ and carbon-RuO_2_ composite diffusion layer for use in direct methanol fuel cells. J. Power Sources 109, 439–445 (2002).

[b59] Liang Z. X. & Zhao T. S. New DMFC anode structure consisting of Platinum nanowires deposited into a nafion membrane. J. Phys. Chem. C 111, 8128–8134 (2007).

[b60] Greeley J. *et al.* Hydrogen evolution over bimetallic systems: understanding the trends. Chem. Phys. Chem. 7, 1032–1035 (2006). 16557633 10.1002/cphc.200500663

