# Supplementary material for: Three-dimensional Nitrogen-Doped Graphene Supported Molybdenum Disulfide Nanoparticles as an Advanced Catalyst for Hydrogen Evolution Reaction
Source: Sci Rep. 2015 Dec 7;5:17542. doi: 10.1038/srep17542 (PMC4670999; doi:10.1038/srep17542)
Supplement: Supplementary Information [file srep17542-s1.doc]

**Three-dimensional Nitrogen-Doped Graphene Supported Molybdenum Disulfide Nanoparticles as an Advanced Catalyst for Hydrogen Evolution Reaction**

Haifeng Dong^1^,*, Conghui Liu^1^, Haitao Ye^2^, Linping Hu^3^, Bunshi Fugetsu^4^, Wenhao Dai^1^, Yu Cao^1^, Xueqiang Qi^3^, Huiting Lu^1^, Xueji Zhang^1^,*

^1^Beijing Key Laboratory for Bioengineering and Sensing Technology, School of Chemistry & Biological Engineering, University of Science & Technology Beijing, Beijing 100083, P.R. China

^2^School of Engineering and Applied Science, Aston University, Birmingham, B4 7ET, United Kingdom

^3^Chemistry and Chemical Engineering, Chongqing University, No. 174 Shazhengjie, Shaping Ba, Chongqing, 400044, P. R. China,

^4^Laboratory of Nanomedicine, Graduate School of Environmental Earth Science, Hokkaido University, Sapporo 060-0810, Hokkaido, Japan

_____________________________________________________________________

* Correspondence author. Tel. & Fax: +86-10-82375840.

*E-mail address:* hfdong@ustb.edu.cn (H. F. Dong)*,* zhangxueji@ustb.edu.cn (X.J. Zhang)


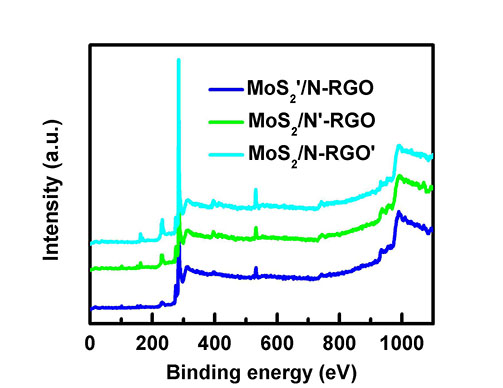


**Figure S1.** The survey XPS spectra of the MoS_2_’/N-RGO, MoS_2_/N’-RGO and MoS_2_/N-RGO’.


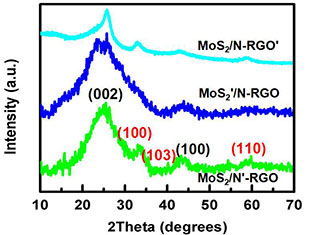


**Figure S2.** XRD patterns of the MoS_2_’/N-RGO, MoS_2_/N’-RGO and MoS_2_/N-RGO’.


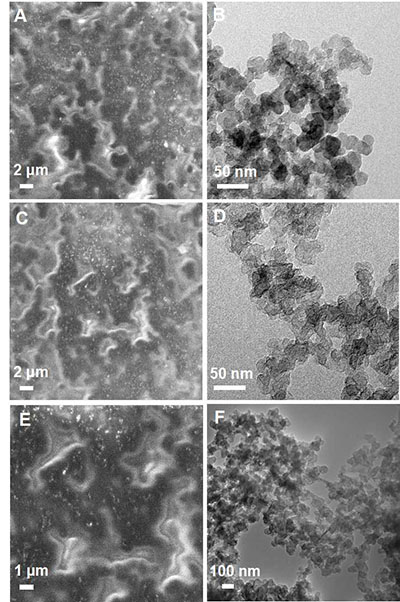


**Figure S3.** (A, C, E) SEM images and (B, D, F) TEM images of the MoS_2_’/N-RGO, MoS_2_/N’-RGO and MoS_2_/N-RGO’.

**
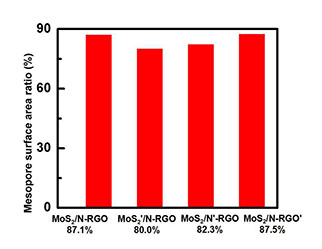
**

**Figure S4.** Mesoporous surface area ratio of the MoS_2_/N-RGO, MoS_2_’/N-RGO, MoS_2_/N’-RGO and MoS_2_/N-RGO’.


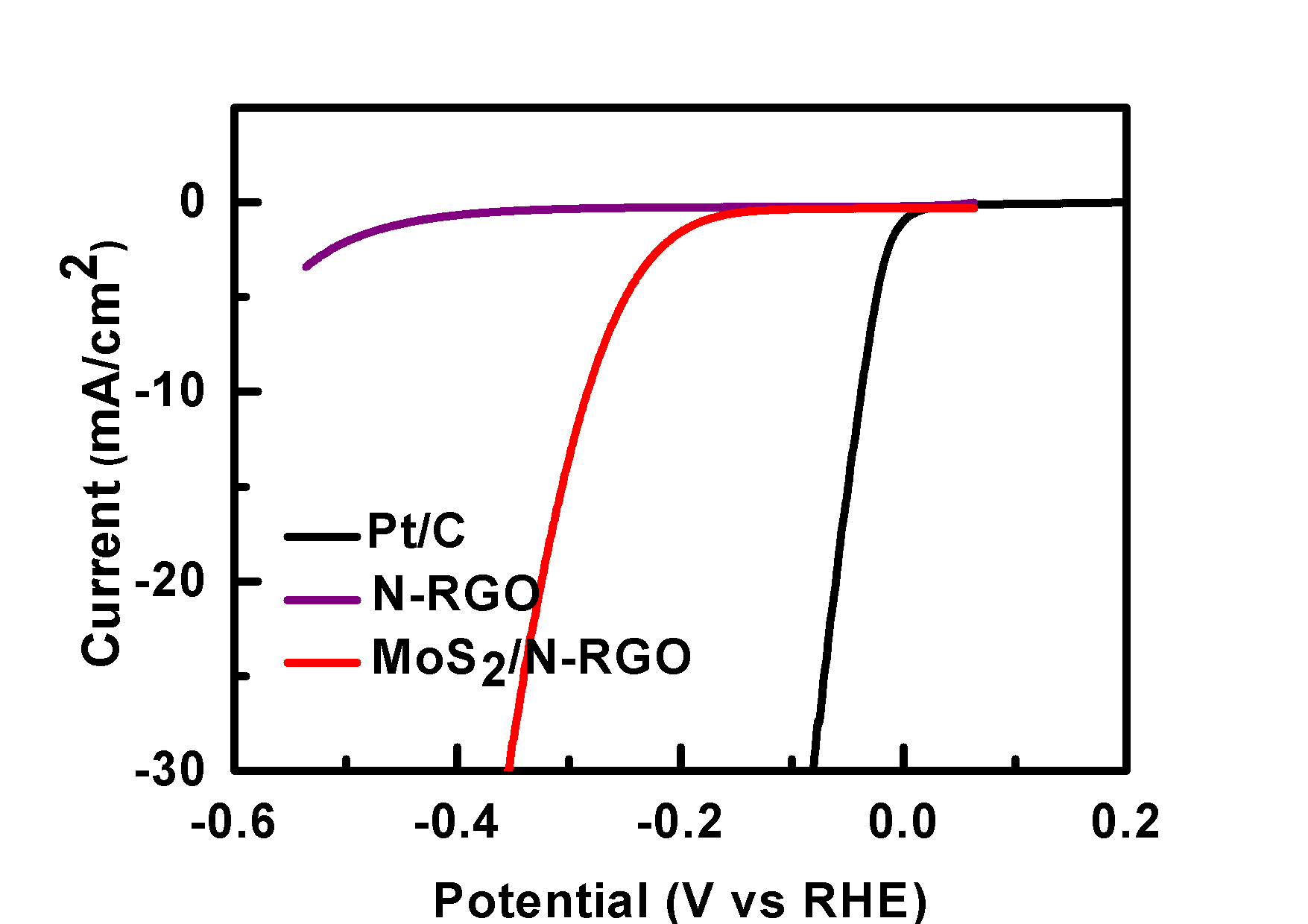


**Figure S5.** Polarization curves of MoS_2_/N-RGO, N-RGO and Pt/C.

**Table S1. XPS Results on Elemental Compositions, the N/C and S**/C Atom Ratios of different catalysts

| **Catalysts** | **C**  **(wt %)** | **O**  **(wt %)** | **N**  **(wt %)** | **Mo**  **(wt %)** | **S**  **(wt %)** | **N/C**  **(at./at.)** | **S/C**  **(at./at.)** |
| --- | --- | --- | --- | --- | --- | --- | --- |
| **MoS_2_/N-RGO** | 78.26 | 7.41 | 5.41 | 5.39 | 3.53 | 0.059 | 0.071 |
| **MoS_2_’/N-RGO** | 86.61 | 4.30 | 4.55 | 3.19 | 1.35 | 0.045 | 0.037 |
| **MoS_2_/N’-RGO** | 80.49 | 5.06 | 5.75 | 5.82 | 2.87 | 0.061 | 0.047 |
| **MoS_2_/N-RGO’** | 77.90 | 5.87 | 7.17 | 5.31 | 3.75 | 0.092 | 0.075 |

**Table S2**. XPS Results on the S Distributions

| **Catalysts** | **S distribution (at. %)** | | | |
| --- | --- | --- | --- | --- |
|  | S 2p_1/2_ | S 2p_3/2_ | S_2_^2-^ | S^4+^ |
| **MoS_2_/N-RGO** | 39 | 42 | 10 | 9 |
| **MoS_2_’/N-RGO** | 34 | 43 | 14 | 9 |
| **MoS_2_/N’-RGO** | 46 | 54 | 0 | 0 |
| **MoS_2_/N-RGO’** | 29 | 22 | 7 | 42 |

**Table S3. XPS Results on the N** Distributions

| **Catalysts** | **N distribution (at. %)** | | | |
| --- | --- | --- | --- | --- |
|  | Nitride-like | Pyridinic N | Pyrrolic N | Graphitic N |
| **MoS_2_/N-RGO** | 47 | 18 | 21 | 14 |
| **MoS_2_’/N-RGO** | 36 | 27 | 18 | 19 |
| **MoS_2_/N’-RGO** | 46 | 25 | 11 | 18 |
| **MoS_2_/N-RGO’** | 45 | 18 | 21 | 14 |
